# Testing the clinical application of the child psychosis-risk screening system (CPSS)

**DOI:** 10.1192/j.eurpsy.2021.1694

**Published:** 2021-08-13

**Authors:** Y. Hamasaki, M. Matsuo, Y. Sakaue, R. Sanada, T. Nakayama, S. Michikoshi, S. Ueba, N. Kurimoto, T. Hikida

**Affiliations:** 1 Faculty Of Contemporary Society, Kyoto Women’s University, kyoto, Japan; 2 Department Of Psychiatry, Shiga University of Medical Science, Shiga, Japan; 3 Department Of Pediatrics, Shiga University of Medical Science, Shiga, Japan; 4 Department Of Pediatrics, Saiseikai Moriyama Municipal Hospital, Shiga, Japan; 5 Department Of Psychiatry, Shigasato Hospital, Shiga, Japan; 6 Institute For Protein Research, Osaka University, Osaka, Japan

**Keywords:** schizophrénia, prodromal state, Screening, CBCL

## Abstract

**Introduction:**

Children in a prodromal state manifesting as truancy or social isolation (hikikomori) often complain of problems that are physical in nature and are subject to significant changes. We developed the Child Psychosis-Risk Screening System (CPSS) that incorporates childhood psycho-behavioral characteristics revealed through a retrospective survey of schizophrenia patients into its algorithm.

**Objectives:**

Our research aimed to test the risk identification of pediatric and psychiatric clinic outpatients using the CPSS.

**Methods:**

We conducted an epidemiological study involving 204 outpatients between the ages of 6 and 14 years who had been examined at a pediatric or psychiatric clinic using the CBCL and clinical data from medical charts. Logistic regression analysis and T-tests were performed using each clinical data variable to clarify the risk of the CPSS calculated from the CBCL data and contributing factors.

**Results:**

The results of the logistic regression analysis demonstrated that the diagnostic category (physical illness or DSM-5 diagnosis) and chief complaint did not contribute to differentiate between the high-risk and low-risk groups. Meanwhile, the environmental factors of “abuse” and “social isolation” did contribute to the discrimination of the two groups.

**Conclusions:**

The fact that the diagnostic category during childhood does not contribute to the discrimination of the high- risk group warrants attention. It is possible that the high-risk group only had a latent endophenotype that had not yet manifested during this period. The factors suggested to have an association with the high-risk group may be reflecting activators and the dynamic state of the critical period for psychosis.

**Disclosure:**

No significant relationships.

